# *Lactobacillus amylovorus* Alleviates *Escherichia coli*-Induced Growth Retardation and Intestinal Dysfunction in Weaning Piglets

**DOI:** 10.3390/ani16142165

**Published:** 2026-07-13

**Authors:** Jianjun Hou, Xuqing Liang, Qiang Li, Xihong Zhou, Guanghui Zhao

**Affiliations:** 1 College of Veterinary Medicine, Northwest A&F University, Xi’an 712100, China; 13709853911@163.com; 2 Institute of Subtropical Agriculture, Chinese Academy of Sciences, Changsha 410125, China; xuqing-liang@stu.hunau.edu.cn; 3 College of Animal Sciences and Technology, Huazhong Agricultural University, Wuhan 430070, China; nwlq19866895@163.com

**Keywords:** diarrhea, *Escherichia coli*, extracellular vesicles, *Lactobacillus amylovorus*, piglets

## Abstract

*Lactobacillus amylovorus* has been reported to inhibit *Escherichia coli* growth in vitro and to modulate intestinal function and microbial composition. However, whether *L. amylovorus* can protect weaning piglets from *E. coli*-induced growth retardation and intestinal dysfunction remains unclear. In the present study, we challenged weaning piglets with *E. coli* K88 and supplemented their diet with *L. amylovorus*. Our results demonstrated that *L. amylovorus* supplementation improved growth performance and reduced diarrhea incidence in *E. coli*-challenged piglets. Furthermore, it enhanced plasma antioxidant capacity and reduced levels of pro-inflammatory cytokines. *L. amylovorus* alleviated *E. coli*-induced villus atrophy and disruption of villus architecture, as evidenced by improved histological morphology. Microbiota profiling showed that *L. amylovorus* improved gut microbial composition, characterized by increased abundance of *Muribaculaceae* and *Lactobacillaceae*. Ileal transcriptome analysis indicated that *L. amylovorus* restored the expression of genes primarily involved in nutrient metabolism. Notably, *L. amylovorus*-derived extracellular vesicles (EVs), but not EV-free supernatant, inhibited *E. coli* growth in vitro. Collectively, these findings suggest that *L. amylovorus* supplementation enhances growth performance, attenuates inflammation, and improves intestinal morphology, function, and microbial homeostasis in *E. coli*-challenged weaning piglets, potentially mediated by its EVs.

## Simple Summary

Pathogenic *Escherichia coli* is a major cause of diarrhea, intestinal damage, and poor growth in weaning piglets, resulting in substantial economic losses in the swine industry. In this study, we investigated whether *L. amylovorus* isolated from fecal samples of Ningxiang pigs could help protect piglets from these problems. We found that dietary supplementation with *L. amylovorus* improved growth performance, reduced diarrhea, enhanced antioxidant capacity, and alleviated intestinal damage and inflammation in piglets challenged with *E. coli*. It also promoted a healthier gut microbial community and restored the expression of genes involved in nutrient metabolism. In addition, extracellular vesicles released by *L. amylovorus* inhibited the growth of *E. coli* in vitro, suggesting a potential mechanism underlying its probiotic effects. These findings indicate that *L. amylovorus* is a promising probiotic candidate for improving intestinal health and reducing the impact of bacterial infections in weaning piglets.

## 1. Introduction

Post-weaning diarrhea (PWD) in piglets is a major cause of economic loss in the swine industry. Among its causative agents, enterotoxigenic *Escherichia coli* (ETEC) is one of the primary contributors, leading to growth retardation, impaired intestinal morphology, heightened inflammatory responses, oxidative stress, and gut microbiota dysbiosis [1,2,3]. Notably, many *E. coli* isolates display resistance to multiple antibiotics, including spectinomycin, apramycin, trimethoprim–sulfonamide, and neomycin [4,5,6], emphasizing the urgent need for effective alternatives in pig production.

Recently, probiotics have emerged as promising alternatives due to their diverse beneficial effects, such as inhibition of pathogen colonization, modulation of immune responses, and maintenance of gastrointestinal homeostasis. Several probiotic strains have been reported to mitigate ETEC-induced PWD in piglets. For example, *Clostridium butyricum* prevents K88-induced diarrhea through metabolites that mediate rectal bacteria–host metabolic cross-talk [7]. *Lactiplantibacillus plantarum* alleviates ETEC-mediated inflammation by inhibiting the NF-κB signaling pathway, enhancing the IgA immune network, and modulating microbial barrier function [8]. Additionally, bacteriocins derived from *L. plantarum* can inhibit DNA replication and interfere with protein synthesis in *E. coli* [9]. A strain of *Lactobacillus amylovorus* isolated from Tibetan pigs has also been shown to suppress *E. coli* growth in vitro and improve intestinal morphology, microbial composition, and carbohydrate metabolism in mice [10]. *L. amylovorus*, as a lactic acid-producing bacterium commonly isolated from the porcine gastrointestinal tract, is well adapted to the anaerobic intestinal environment. In addition, it exhibits strong lactic acid production and generates antimicrobial metabolites that may contribute to the competitive exclusion of pathogenic bacteria.

However, it remains unclear whether *L. amylovorus* can protect weaning piglets from ETEC-induced intestinal damage in vivo, and the underlying mechanisms are largely unknown. Therefore, in the present study, we investigated the effects of *L. amylovorus* isolated from Ningxiang pigs on growth performance, inflammatory responses, intestinal morphology, and gut microbiota composition in ETEC-challenged weaning piglets. Moreover, we explored the potential mechanisms by which *L. amylovorus* exerts protective effects. Our findings aim to provide scientific evidence supporting the use of *L. amylovorus* as a potential alternative to antibiotics in the management of PWD in weaning piglets.

## 2. Materials and Methods

### 2.1. Ethics Statement

All experimental protocols were reviewed and approved by the Protocol Management and Review Committee at the Institute of Subtropical Agriculture, Chinese Academy of Sciences (Approval number: 20250414).

### 2.2. Animals and Experimental Design

A total of 24 healthy male piglets, weaned at 21 days of age, were selected for this study. All piglets were obtained from four litters (six piglets per litter) and were randomly allocated to the three experimental groups to minimize potential litter effects. All animals were kept in individual pens. Following a 3-day acclimation period, the piglets with an initial body weight of approximately 7.90 kg were randomly assigned to three groups (*n* = 8 per group): (1) a control group fed a basal diet (CON); (2) a group fed a basal diet and challenged with *E. coli* (ECO); and (3) a group fed a basal diet supplemented with *L. amylovorus* (5 g/kg feed) and challenged with *E. coli* (LAM). *L. amylovorus* was cultured, freeze-dried into a bacterial powder (10^9^ CFU/g), and then thoroughly mixed with the basal diet. The experimental diet was formulated based on a corn–soybean meal diet (Supplementary Table S1) to meet the nutrient requirements of pigs, according to NRC (2012) [11]. Starting on day 8 of the experiment, piglets in the ECO and LAM groups were orally administered 10 mL of *E. coli* K88 suspension (10^9^ CFU/mL) per day for three consecutive days. *L. amylovorus* was isolated from fecal samples of Ningxiang pigs and deposited in the China Center for Type Culture Collection (CCTCC) under accession number M2024555, and it was supplemented throughout the entire experiment. Prior to the experiment, the pig facility was thoroughly disinfected, and feeders and drinkers were cleaned. Throughout the 14-day experimental period, piglets were fed four times daily (08:00, 12:00, 16:00, and 20:00), with feed provided ad libitum to ensure slight leftovers in the trough. All groups were maintained under identical management conditions. All animals were housed individually in pig pens, which were kept at approximately 25 °C with adequate ventilation, cleanliness, and dryness. Routine disinfection was performed following standard procedures. Piglets were monitored daily for behavior and feed intake, and any abnormalities were promptly recorded and addressed. No mortality occurred throughout the experimental period.

### 2.3. Growth Performance and Diarrhea Frequency

The body weight (BW) of each piglet was measured individually at the beginning and at the end of the experimental period. Feed intake was recorded daily for each pen, and the average daily feed intake (ADFI) per piglet was calculated. The feed-to-gain ratio (F:G) was determined as the ratio of total feed intake to body weight gain over the experimental period. Piglets were observed twice daily for signs of diarrhea, which was scored based on fecal consistency using a standard scoring system: 0 = normal, 1 = soft feces, 2 = mild diarrhea, and 3 = severe diarrhea [12]. The incidence of diarrhea in each group was calculated using the following formula: Diarrhea incidence (%) = (Number of piglets with diarrhea)/(Total number of piglets) × 100. All measurements and observations were conducted by trained personnel under standardized management conditions throughout the experimental period.

### 2.4. Plasma Biochemical Parameter Determination

At the end of the experimental period, blood samples were collected from the jugular vein of each piglet into heparinized tubes. Plasma was separated by centrifugation at 3000× *g* for 10 min at 4 °C and stored at −80 °C until analysis. Plasma biochemical parameters, including alanine aminotransferase (ALT), aspartate aminotransferase (AST), alkaline phosphatase (ALP), Urea, and creatinine (Cr), were measured using an automated biochemical analyzer (Hitachi 7020, Hitachi High-Tech Corporation, Tokyo, Japan) following the manufacturer’s instructions. All measurements were performed in duplicate, and quality control standards were included to ensure the accuracy and precision of the assays.

### 2.5. Determination of Plasma Antioxidant-Related Parameters

Plasma antioxidant-related parameters, including the activities of glutathione peroxidase (GSH-Px; Cat. No. A005-1-2) and superoxide dismutase (SOD; Cat. No. A001-1-2), total antioxidant capacity (T-AOC; Cat. No. A015-2-1), and malondialdehyde (MDA; Cat. No. A003-4-1) content, were measured using commercial kits (Jiancheng, Nanjing, China) according to the manufacturers’ instructions.

### 2.6. Determination of Plasma Inflammatory Cytokine Concentration

Plasma concentrations of inflammatory cytokines, including tumor necrosis factor-alpha (TNF-α; Cat. No. BYHS500565) and interleukin-1 beta (IL-1β; Cat. No. BY-EP771042), were determined using commercial kits (Boyan, Nanjing, China) following the manufacturers’ instructions.

### 2.7. Histological Analysis of Ileal Morphology

At the end of the experimental period, all piglets were fasted for 8 h and then euthanized. An approximately 2–3 cm segment of the distal ileum was collected from each piglet, gently rinsed with PBS to eliminate intestinal contents and subsequently fixed in 10% neutral-buffered formalin for 48 h. The fixed samples were routinely processed for histological examination, including ethanol dehydration, xylene clarification, paraffin embedding, and preparation of 5 μm sections. Tissue sections were subjected to H&E staining and observed using an Olympus BX53 light microscope (Olympus Corporation, Tokyo, Japan). For morphometric evaluation, 10 intact villi and their corresponding crypts were randomly chosen from each section. Villus height (VH) was measured from the apex of the villus to the villus–crypt interface, while crypt depth (CD) was recorded from this interface to the crypt base. The VH/CD ratio was then calculated. Quantitative measurements were obtained using Image-Pro Plus 6.0 software (Media Cybernetics, Rockville, MD, USA), with all analyses performed by a blinded evaluator.

### 2.8. Gut Microbiota Profiling

Fresh fecal samples were collected immediately after spontaneous defecation from live piglets prior to euthanasia at the end of the experimental period, immediately frozen in liquid nitrogen, and stored at −80 °C until analysis. Total microbial DNA was extracted using a Stool DNA Kit (TianGen, Beijing, China). The V3–V4 region of the bacterial 16S rRNA gene was amplified using universal primers (forward: 341F, reverse: 806R). PCR products were purified, quantified, and pooled in equimolar concentrations to construct the sequencing library. High-throughput sequencing was performed by Beijing Novogene Bioinformatics Technology Co., Ltd. (Beijing, China) on the Illumina NovaSeq X Plus platform to generate paired-end reads. Raw sequences were subjected to quality control, including adaptor trimming, low-quality read filtering, and chimera removal. Clean reads were clustered into operational taxonomic units (OTUs) at 97% sequence similarity using QIIME 2. Alpha and beta diversity metrics, as well as microbial composition analyses, were performed using the standard bioinformatics pipelines (Novogene, Beijing, China).

### 2.9. Ileal Transcriptome Analysis

Ileal samples were harvested at the end of the experiment, immediately frozen in liquid nitrogen, and stored at −80 °C. Total RNA was extracted with TRIzol reagent, followed by quality assessment using a NanoDrop spectrophotometer (Thermo Fisher Scientific, Wilmington, DE, USA) and an Agilent 2100 Bioanalyzer (Agilent Technologies, Santa Clara, CA, USA). Libraries were generated with the NEBNext Ultra RNA Library Prep Kit (New England Biolabs, Ipswich, MA, USA) and sequenced on an Illumina NovaSeq platform. Following quality filtering, reads were aligned to the *Sus scrofa* reference genome (Sscrofa11.1) using HISAT2 (v2.2.1). Transcript abundance was quantified by StringTie, and differentially expressed genes were determined with DESeq2 (|log_2_ fold change| ≥ 1, adjusted *p* < 0.05). GO and KEGG enrichment analyses were conducted to identify significantly enriched biological functions and pathways.

### 2.10. Quantitative Reverse Transcription PCR (RT-qPCR) Analysis

Frozen ileal tissues were used for total RNA extraction with TRIzol reagent (Invitrogen, Shanghai, China). Complementary DNA was synthesized using a commercial reverse transcription kit (Applied Biosystems, Shanghai, China), and gene expression was quantified by real-time PCR with SYBR chemistry on a Roche LightCycler 480 II system (Indianapolis, IN, USA). Transcript levels were normalized against β-actin and expressed using the 2^−ΔΔCt^ method. Primer information is provided in Supplementary Table S2.

### 2.11. EVs Characterization by Transmission Electron Microscopy (TEM) and Nanoparticle Tracking Analysis (NTA)

*L. amylovorus* was cultured in MRS broth at 37 °C for 18–24 h. EVs were then isolated from the bacterial culture. Briefly, the conditioned medium was collected and sequentially centrifuged at 1000× *g*, 3000× *g*, and 10,000× *g* for 30 min each at 4 °C to remove cells and debris. The resulting supernatant was filtered through a 0.22 μm membrane to eliminate residual bacteria and large particles. The filtrate was subsequently ultracentrifuged at 150×, 1000× *g* for 3 h at 4 °C to obtain EVs. The morphology of the isolated EVs was examined using TEM (Hitachi HT-7700, Hitachi High-Tech Corporation, Tokyo, Japan). Size distribution and particle concentration (diluted 20,000-fold) were analyzed using NTA with a ZetaView instrument (Particle Metrix, Munich, Germany).

### 2.12. Antibacterial Properties of L. amylovorus

The antibacterial activity of *L. amylovorus*-derived EVs and EV-free supernatant (EFS) was evaluated using the agar well diffusion method by measuring the diameter of inhibition zones. Briefly, an *E. coli* suspension was evenly spread onto Müller–Hinton agar plates. Wells of 6 mm diameter were aseptically punched into the agar. Subsequently, 100 μL of prepared EVs (100 μg protein, 10^9^ particles per μg protein) or EFS was added into each well under aseptic conditions. Gentamicin sulfate served as the positive control, and sterile culture medium was used as the negative control. Following 24 h of incubation at 37 °C, inhibition halos were measured with a digital caliper. All measurements were obtained from three independent replicates.

### 2.13. Statistical Analysis

Statistical analyses were conducted using SPSS Statistics (version 22.0). Differences among groups were assessed by one-way ANOVA followed by the Student–Newman–Keuls multiple comparison test. Data are presented as mean ± SEM, and statistical significance was defined as *p* < 0.05.

## 3. Results

### 3.1. L. amylovorus Alleviated E. coli-Induced Growth Retardation and Diarrhea in Weaning Piglets

**Figure 1:**
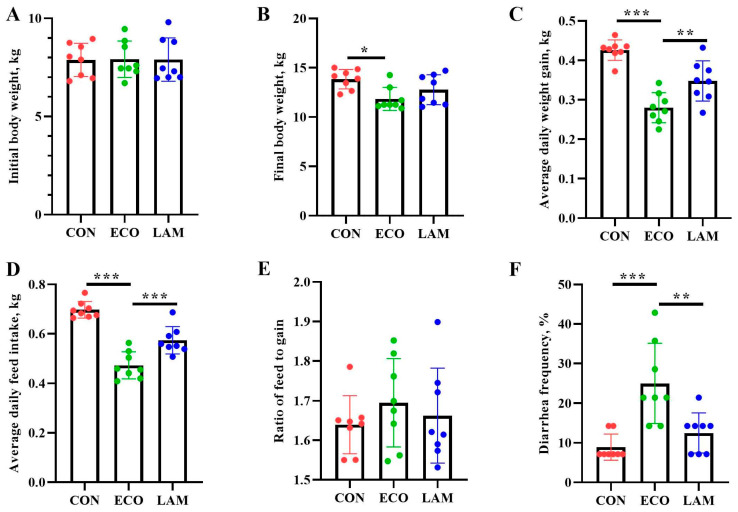
*L. amylovorus* alleviated *E. coli*-induced growth retardation and diarrhea in weaning piglets. (**A**) Initial body weight; (**B**) final body weight; (**C**) average daily weight gain; (**D**) average daily feed intake; (**E**) ratio of feed to gain; (**F**) diarrhea frequency. CON, piglets fed a basal diet; ECO, piglets fed a basal diet and challenged with *E. coli*; LAM, piglets fed a basal diet supplemented with *L. amylovorus* and challenged with *E. coli*. Data were presented as Mean ± SEM, *n* = 8. * *p* < 0.05; ** *p* < 0.01; *** *p* < 0.001.

Healthy weaned piglets, with an initial body weight of approximately 7.90 kg at 24 days of age, were randomly assigned to three treatment groups, with no significant differences in initial body weight among the groups (Figure 1A). At the end of the experiment, control piglets exhibited a significantly higher final body weight compared with those challenged with *E. coli* (Figure 1B). Both the control group and the group supplemented with *L. amylovorus* showed higher average daily gain (ADG) and ADFI than the *E. coli*-challenged group (Figure 1C,D). No significant differences in the feed-to-gain ratio (F: G) were observed among the three groups (Figure 1E). Additionally, control piglets and those receiving *L. amylovorus* supplementation had a lower frequency of diarrhea compared with the *E. coli*-challenged group (Figure 1F).

### 3.2. L. amylovorus Improved Plasma Biochemical Parameter in E. coli-Challenged Piglets

**Figure 2:**
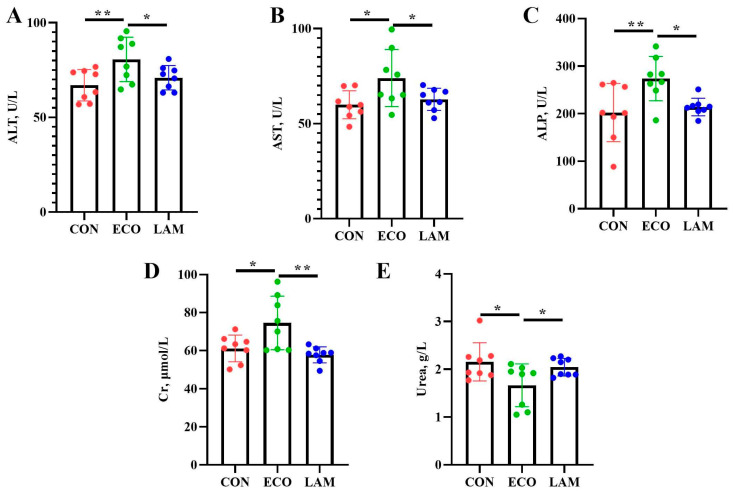
*L. amylovorus* improved plasma biochemical parameters in *E. coli*-challenged piglets. (**A**) Plasma ALT activity; (**B**) plasma AST activity; (**C**) plasma ALP activity; (**D**) plasma Cr content; (**E**) plasma urea content. ALT, alanine aminotransferase; AST, aspartate aminotransferase; ALP, alkaline phosphatase; Cr, creatinine. CON, piglets fed a basal diet; ECO, piglets fed a basal diet and challenged with *E. coli*; LAM, piglets fed a basal diet supplemented with *L. amylovorus* and challenged with *E. coli*. Data were presented as Mean ± SEM, *n* = 8. * *p* < 0.05; ** *p* < 0.01.

As shown in Figure 2, *E. coli* challenge significantly increased plasma activities of ALT (Figure 2A), AST (Figure 2B), and ALP (Figure 2C), as well as Cr levels (Figure 2D), while decreasing urea levels (Figure 2E). Supplementation with *L. amylovorus* significantly mitigated these changes, restoring the biochemical parameters toward those observed in the control group.

### 3.3. L. amylovorus Alleviated Oxidative Stress and Inflammatory Response in E. coli-Challenged Piglets

**Figure 3:**
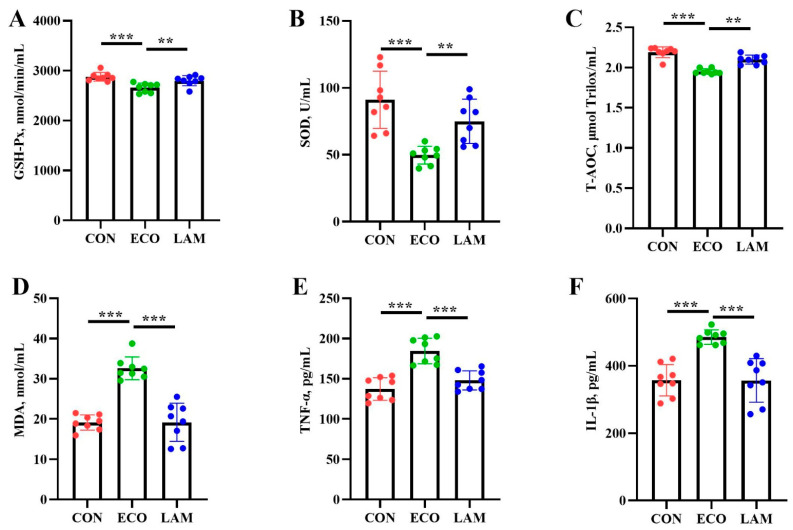
*L. amylovorus* alleviated oxidative stress and inflammatory response in *E. coli*-challenged piglets. (**A**) Plasma GSH-Px activity; (**B**) plasma SOD activity; (**C**) plasma T-AOC activity; (**D**) plasma MDA content; (**E**) plasma TNF-α content; (**F**) plasma IL-1β content. CON, piglets fed a basal diet; ECO, piglets fed a basal diet and challenged with *E. coli*; LAM, piglets fed a basal diet supplemented with *L. amylovorus* and challenged with *E. coli*. Data were presented as Mean ± SEM, *n* = 8. ** *p* < 0.01; *** *p* < 0.001.

*E. coli* challenge significantly decreased plasma activities of GSH-Px (Figure 3A), SOD (Figure 3B), and T-AOC (Figure 3C), while increasing the levels of MDA (Figure 3D), TNF-α (Figure 3E), and IL-1β (Figure 3F). Supplementation with *L. amylovorus* significantly attenuated these alterations, restoring the parameters toward levels observed in the control group.

### 3.4. L. amylovorus Alleviated Morphology Impairment in E. coli-Challenged Piglets

**Figure 4:**
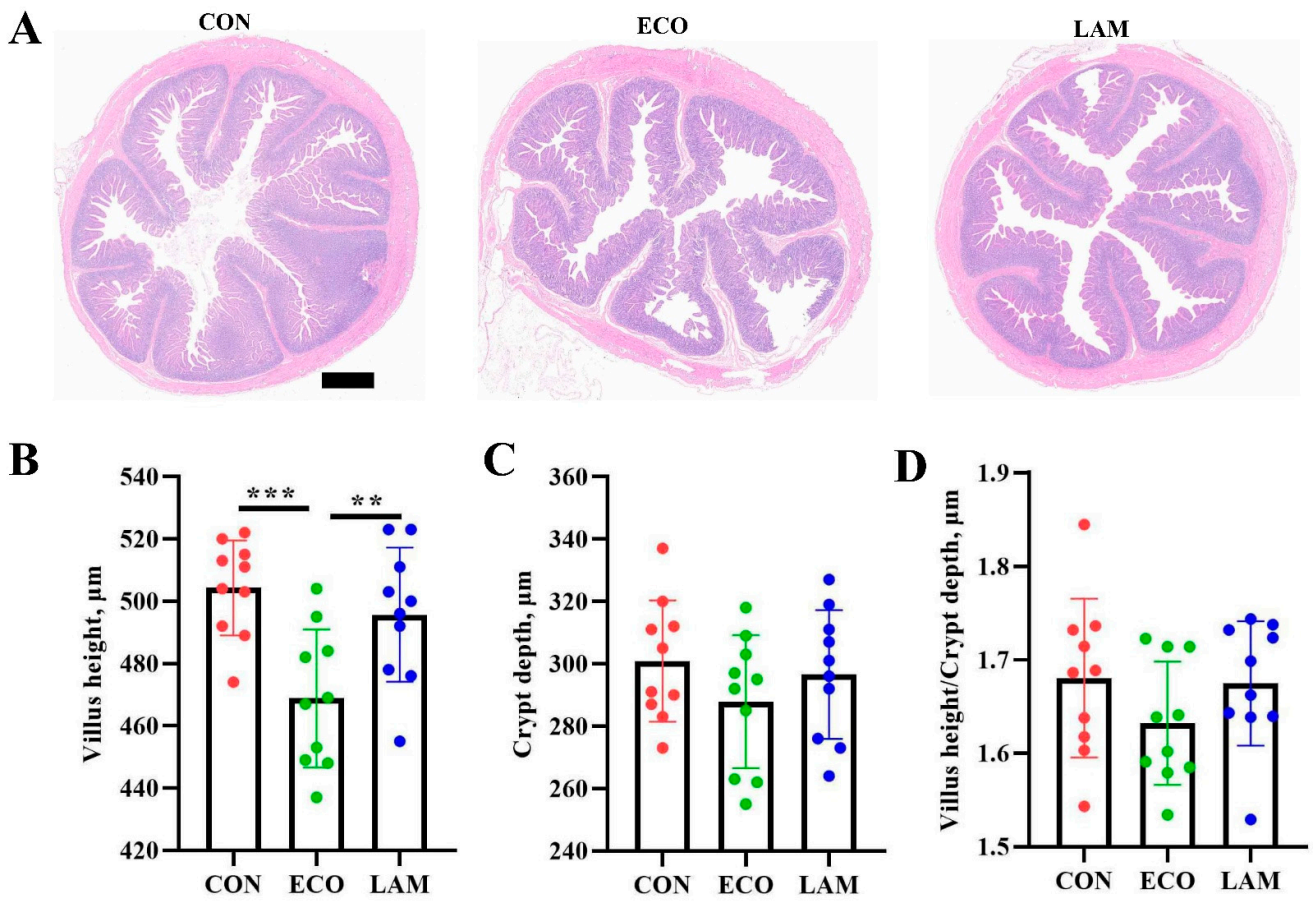
*L. amylovorus* alleviated morphology impairment in *E. coli*-challenged piglets. (**A**) Ileal morphology (Scale bar = 1.0 mm); (**B**) villus height; (**C**) crypt depth; (**D**) the ratio of villus height to crypt depth; CON, piglets fed a basal diet; ECO, piglets fed a basal diet and challenged with *E. coli*; LAM, piglets fed a basal diet supplemented with *L. amylovorus* and challenged with *E. coli*. Data were presented as Mean ± SEM, *n* = 10. ** *p* < 0.01; *** *p* < 0.001.

*E. coli* challenge induced villus atrophy and disrupted villus architecture in the ileum, whereas supplementation with *L. amylovorus* significantly alleviated these alterations (Figure 4A). Both the control group and the group receiving *L. amylovorus* supplementation exhibited greater villus height compared with the *E. coli*-challenged group (Figure 4B). No significant differences were observed among the three groups in crypt depth or the villus height-to-crypt depth ratio (Figure 4C,D).

### 3.5. L. amylovorus Improved Gut Microbiota Composition in E. coli-Challenged Piglets

**Figure 5:**
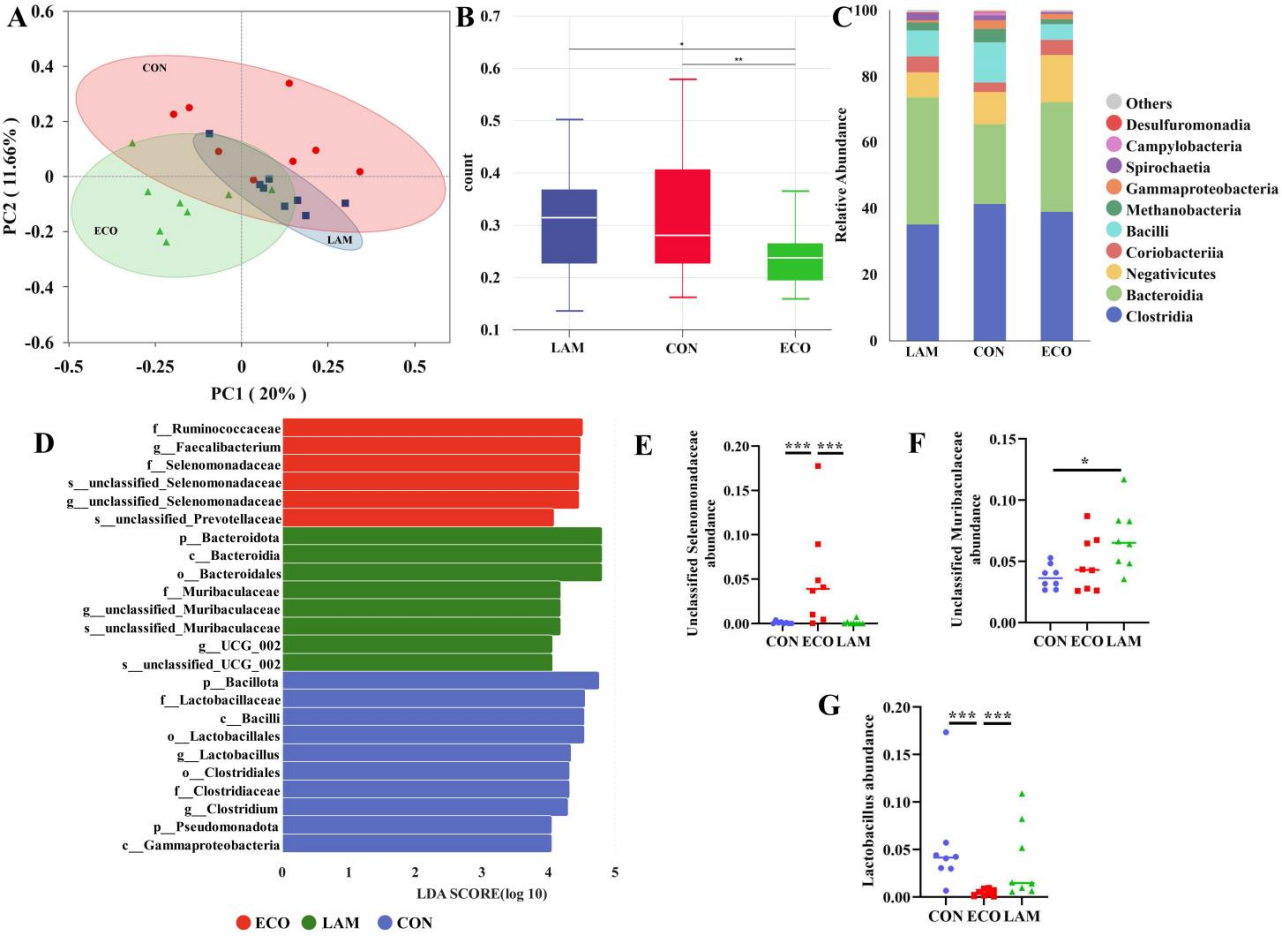
*L. amylovorus* improved gut microbiota composition in *E. coli*-challenged piglets. (**A**) PCoA results; (**B**) difference in beta diversity; (**C**) microbiota composition at the class level; (**D**) LEfSe results; relative abundance of *Selenomonadaceae* (**E**), *Muribaculaceae* (**F**), and Lactobacillus (**G**). CON, piglets fed a basal diet; ECO, piglets fed a basal diet and challenged with *E. coli*; LAM, piglets fed a basal diet supplemented with *L. amylovorus* and challenged with *E. coli*. Data were presented as Mean ± SEM, *n* = 8. * *p* < 0.05; ** *p* < 0.01; *** *p* < 0.001.

Principal coordinate analysis (PCoA) of gut microbiota revealed a clear separation between the gut microbial communities of the *E. coli*-challenged (ECO) piglets and the control (CON) piglets, whereas the microbial community of the *L. amylovorus*-supplemented (LAM) group was positioned between the ECO and CON groups (Figure 5A). Beta diversity analysis based on weighted UniFrac distances further demonstrated significant differences in microbial community structure between the LAM and ECO groups, as well as between the CON and ECO groups (Kruskal–Wallis test, *p* < 0.05) (Figure 5B). At the class level, Clostridia, Bacteroidia, and Negativicutes were the dominant bacterial taxa in the gut microbiota (Figure 5C). Compared with the CON and LAM groups, piglets in the ECO group exhibited a higher relative abundance of Negativicutes. LEfSe analysis identified *Selenomonadaceae* as the characteristic taxon in the ECO group, *Muribaculaceae* in the LAM group, and *Lactobacillaceae* in the CON group (Figure 5D). Further analysis revealed that the relative abundance of *Selenomonadaceae* was significantly higher in the ECO group than in the other two groups (Figure 5E), whereas *Muribaculaceae* was significantly enriched in the LAM group compared with the CON group (Figure 5F). In addition, the relative abundance of *Lactobacillaceae* was significantly reduced in the ECO group compared with the CON and LAM groups (Figure 5G).

### 3.6. L. amylovorus Regulated Ileal Gene Expression in E. coli-Challenged Piglets

**Figure 6:**
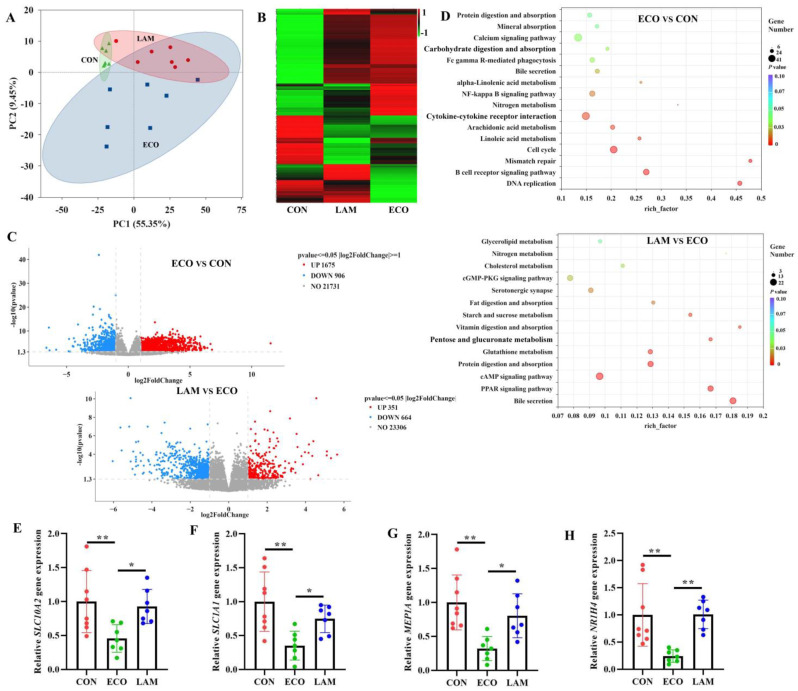
*L. amylovorus* regulated ileal gene expression in *E. coli*-challenged piglets. (**A**) PCoA result; (**B**) Heatmap result; (**C**) Volcano result; (**D**) KEGG result; Relative gene expression of *SLC10A3* (**E**), *SLC1A1* (**F**), *MEP1A* (**G**), and *NR1H4* (**H**). CON, piglets fed a basal diet; ECO, piglets fed a basal diet and challenged with *E. coli*; LAM, piglets fed a basal diet supplemented with *L. amylovorus* and challenged with *E. coli*. Data were presented as Mean ± SEM, *n* = 7. * *p* < 0.05; ** *p* < 0.01.

Ileal gene expression profiles were analyzed by transcriptome sequencing. PCoA revealed a clear separation between the ECO and control groups, whereas the LAM group was positioned between the ECO and CON groups (Figure 6A). These findings were further supported by the gene expression heatmap (Figure 6B). Volcano plot analysis showed that, compared with the CON group, 1675 genes were significantly upregulated and 906 genes were significantly downregulated in the ECO group. In addition, compared with the ECO group, 351 genes were significantly upregulated and 664 genes were significantly downregulated in the LAM group (Figure 6C). KEGG pathway enrichment analysis demonstrated that the differentially expressed genes (DEGs) between the ECO and CON groups were mainly associated with nutrient metabolism pathways, including protein digestion and absorption, bile secretion, and nitrogen metabolism, as well as inflammatory signaling pathways such as the NF-κB signaling pathway and B cell receptor signaling pathway (Figure 6D). Furthermore, DEGs between the LAM and ECO groups were primarily enriched in nutrient metabolism-related pathways, including protein digestion and absorption, bile secretion, and nitrogen metabolism. Notably, several genes involved in nutrient metabolism and intestinal function, including solute carrier family 10 member 2 (*SLC10A2*) (Figure 6E), *SLC1A1* (Figure 6F), meprin A subunit alpha (*MEP1A*) (Figure 6G), and nuclear receptor subfamily 1 group H member 4 (*NR1H4*) (Figure 6H), were significantly downregulated following *E. coli* challenge, whereas *L. amylovorus* supplementation markedly restored their expression levels.

### 3.7. L. amylovorus-Derived EVs Exhibited Antibacterial Activity Against E. coli

**Figure 7:**
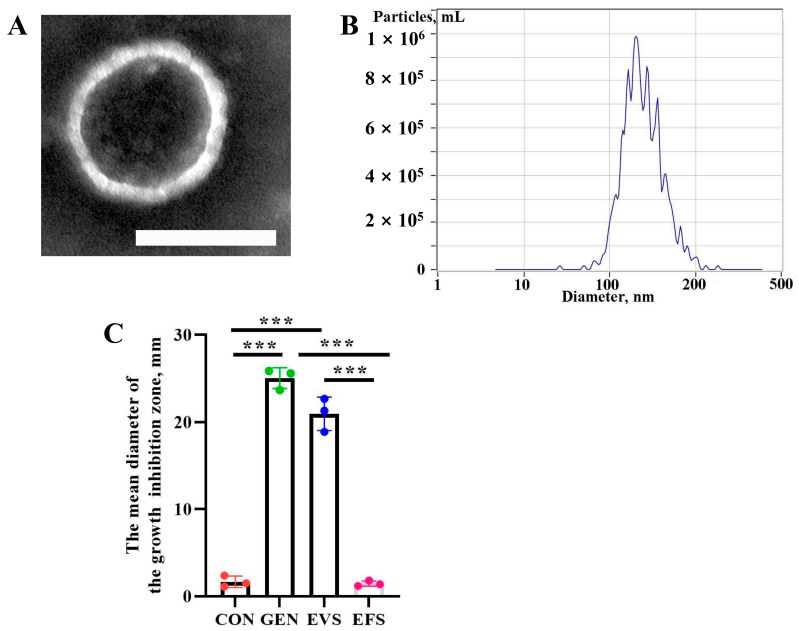
*L. amylovorus*-derived EVs exhibited antibacterial activity against *E. coli.* (**A**) EVs characterization by transmission electron microscopy (scale bar = 100 nm); (**B**) nanoparticle tracking analysis (the sample was diluted 20,000-fold before analysis); (**C**) the mean diameter of the growth inhibition zone. CON, *E. coli* treated with sterile culture medium; GEN, *E. coli* treated with gentamicin sulfate; EVS, *E. coli* treated with *L. amylovorus*-derived EVs; EFS, *E. coli* treated with EVs-free supernatant. Data were presented as Mean ± SEM, *n* = 3. *** *p* < 0.001.

*L. amylovorus*-derived EVs were isolated and characterized by TEM, which revealed typical spherical vesicular structures (Figure 7A). NTA showed that the EV concentration was 6.0 × 10^11^ particles/mL, with a mean particle diameter of approximately 130 nm (Figure 7B). Antibacterial activity assays demonstrated that *L. amylovorus*-derived EVs exhibited inhibitory effects against *E. coli* comparable to those of gentamicin (Figure 7C). In contrast, the EV-free supernatant showed no detectable antibacterial activity against *E. coli*.

## 4. Discussion

Among the most widely studied probiotics, *Lactobacillus* species are commonly used for the prevention and treatment of various diseases, with *L. amylovorus* exhibiting strong resistance to gastrointestinal conditions and exerting multiple beneficial effects. Notably, *L. amylovorus* has been shown to inhibit the growth of *E. coli* in vitro and alleviate *E. coli*-induced inflammation in mice [10,13]. In addition, *L. amylovorus* has been reported to suppress excessive production of inflammatory cytokines, restore gut microbiota balance, improve carbohydrate metabolism and lactose utilization, and enhance intestinal barrier function [10,14,15,16]. Both the bacteria themselves and their cell-free supernatants have demonstrated anti-inflammatory effects [13]. Importantly, the beneficial effects of *L. amylovorus* are suggested to be mediated, at least in part, by its production of short-chain fatty acids, enzymes, and EVs [10,14,17]. In the present study, we further confirmed that *L. amylovorus* improves growth performance, modulates inflammatory responses, preserves intestinal morphology, and maintains microbiota composition in weanling piglets challenged with *E. coli*. Furthermore, we found that *L. amylovorus*-derived EVs, but not the cell-free supernatant, effectively inhibited the growth of *E. coli*, highlighting a potential key role of EVs in mediating its antibacterial activity.

Plasma biochemical markers, including ALT, AST, ALP, Cr, and Urea, are key indicators of liver and kidney function as well as overall metabolic health. In the present study, *E. coli* challenge significantly elevated ALT and AST activities, indicating hepatocellular stress or mild liver injury, which is consistent with previous reports showing that pathogenic *E. coli* can induce systemic inflammation and hepatic impairment [18]. Similarly, the increase in ALP suggests potential cholestatic stress or altered liver metabolism. Elevated Cr and Urea levels observed in challenged piglets reflect impaired renal excretory function or increased protein catabolism under infection-induced stress [19]. Importantly, supplementation with *L. amylovorus* effectively mitigated these alterations, normalizing ALT, AST, ALP, Cr, and Urea levels. This protective effect likely results from a combination of improved gut barrier function, modulation of inflammatory responses, and stabilization of nutrient metabolism, highlighting the capacity of *L. amylovorus* to alleviate hepatic and renal stress associated with *E. coli* infection in weaning piglets. These findings support the possible role of probiotic intervention in maintaining systemic homeostasis during enteric bacterial challenges.

We found that *E. coli* challenge induced marked oxidative stress and intestinal injury in weaning piglets. Activities of key antioxidant enzymes were significantly reduced, while MDA level was elevated, indicating enhanced lipid peroxidation and compromised antioxidant defenses. Concurrently, plasma pro-inflammatory cytokines, IL1β and TNFα, were significantly increased, reflecting a systemic inflammatory response consistent with previous reports on ETEC infection [20,21]. Histological analysis further revealed villus atrophy and disrupted villus architecture in the ileum, suggesting impaired intestinal barrier integrity and reduced absorptive capacity. Supplementation with *L. amylovorus* effectively alleviated these alterations by enhancing antioxidant enzyme activities, reducing MDA accumulation, suppressing pro-inflammatory cytokine production, and restoring villus height and organization. Collectively, our findings highlight the protective role of *L. amylovorus* against pathogen-induced oxidative and inflammatory damage, contributing to the maintenance of intestinal structure and function and promoting improved growth performance in weaning piglets.

Gut microbiota profiling revealed that *E. coli* challenge disrupted the intestinal microbial community in weaning piglets, consistent with previous studies reporting that enterotoxigenic *E. coli* infection induces dysbiosis and reduces microbial diversity [22,23]. LEfSe analysis showed an enrichment of *Selenomonadaceae* in *E. coli*-challenged piglets, suggesting a potential association with pathogen-induced intestinal imbalance and inflammation [24]. In contrast, *Muribaculaceae*, a bacterial family associated with the alleviation of inflammation [25], was enriched in piglets supplemented with *L. amylovorus*, indicating that *L. amylovorus* may promote the enrichment of beneficial microbial taxa. The predominance of *Lactobacillaceae* in control piglets aligns with their well-established roles in promoting epithelial barrier integrity, generating beneficial short-chain fatty acids, and regulating mucosal immune responses [26]. Collectively, these findings indicate that *L. amylovorus* supplementation can partially restore the gut microbiota disrupted by *E. coli*, supporting its role in maintaining intestinal homeostasis and mitigating pathogen-induced dysbiosis.

Ileal transcriptome analysis revealed that *E. coli* challenge substantially altered the gene expression landscape in weaning piglets, whereas supplementation with *L. amylovorus* partially restored these transcriptional changes. Among those DEGs, *SLC10A2*, *SLC1A1*, *MEP1A*, and *NR1H4* were prominently affected by *E. coli* infection and subsequently modulated by *L. amylovorus*, suggesting a key role in intestinal nutrient handling and metabolic homeostasis. SLC10A2 encodes the apical sodium-dependent bile acid transporter, which is essential for bile acid reabsorption and enterohepatic circulation, and its dysregulation has been associated with impaired lipid and nutrient absorption following intestinal injury [27]. SLC1A1, a glutamate transporter, participates in amino acid uptake and neuronal signaling in the gut–brain axis, with reduced expression linked to disrupted nutrient utilization under inflammatory conditions [28]. MEP1A encodes meprin A, a metalloprotease involved in extracellular matrix remodeling and intestinal barrier maintenance, and its downregulation has been observed in models of colitis and enteric infection [29]. NR1H4, also known as the farnesoid X receptor (FXR), functions as a bile acid–activated transcription factor that coordinates bile acid metabolism, glucose homeostasis, and anti-inflammatory responses [30]. The restoration of these genes by *L. amylovorus* suggests that probiotic supplementation may preserve nutrient transport, bile acid signaling, and mucosal integrity, thereby mitigating the metabolic disruptions induced by *E. coli*. These transcriptional adaptations are consistent with improved intestinal function and host resilience observed in probiotic-treated animals, highlighting a mechanistic link between microbial modulation and host metabolic regulation.

It is well established that the beneficial effects of *Lactobacillus* species are primarily mediated by their metabolites, including short-chain fatty acids, bacteriocins, and other bioactive compounds that influence host physiology and modulate microbial ecology [31]. More recently, EVs have emerged as critical mediators of host–microbe interactions, facilitating the transfer of microbial signals, proteins, lipids, and nucleic acids to host cells, thereby modulating immune responses and enhancing intestinal barrier function [32]. A previous study demonstrated that *L. amylovorus*-derived EVs contribute to intestinal barrier maturation and immune programming during early life in neonatal piglets, highlighting their key role in gut development [17]. In the present study, we further showed that *L. amylovorus*-derived EVs significantly inhibited the growth of *E. coli* in vitro, confirming the pivotal role of bacteria-derived EVs in antimicrobial defense.

## 5. Conclusions

Our study demonstrates that *L. amylovorus* supplementation alleviates the adverse effects of *E. coli* challenge, including impaired growth performance, intestinal inflammation, and gut microbiota dysbiosis, in weaning piglets. Furthermore, our findings suggest that *L. amylovorus*-derived EVs may represent a functional component contributing to the probiotic’s antibacterial activity. Nevertheless, their in vivo efficacy and underlying mechanisms remain to be established. Further studies are required to determine whether these EVs can recapitulate the beneficial effects of the live probiotic and to evaluate their potential as post-biotic agents for preventing enteric infections.

## Data Availability

The 16S rRNA and scRNA-seq raw data have been deposited in the Genome Sequence Archive of the National Genomics Data Center, China National Center for Bioinformation (Beijing), under accession number PRJCA065296, and are publicly accessible at https://ngdc.cncb.ac.cn/gsa (accessed on 1 June 2026).

## Supplementary Materials

The following supporting information can be downloaded at: https://www.mdpi.com/article/10.3390/ani16142165/s1, Table S1: Diet formulation and calculated nutrient values; Table S2: Primer sequences for RT-qPCR.
