# Supplementary material for: Lactobacillus amylovorus Alleviates Escherichia coli-Induced Growth Retardation and Intestinal Dysfunction in Weaning Piglets
Source: Animals (Basel). 2026 Jul 13;16(14):2165. doi: 10.3390/ani16142165 (PMC13405008; doi:10.3390/ani16142165)
Supplement: Supplementary file 1 [file animals-16-02165-s001.zip › animals-4394460-supplementary.pdf]

Supplementary Table S1 Diet formulation and calculated nutrient values

Diet component	%	Calculated nutrient value	
Corn	58.0	Digestible Energy, MC/kg	3.48
Soybean meal	12.0	Crude protein, %	17.4
Extruded soybean	10.0	Ca, %	0.69
Fish meal	3.0	Total P, %	0.63
Whey powder	9.0	Digestible P, %	0.40
Fat powder	2.0	Digestible lysine, %	1.57
Dicalcium phosphate	0.7	Digestible methionine, %	0.44
Limestone	0.4	Digestible threonine, %	0.96
NaCl	0.4	Digestible tryptophan, %	0.24
L-lysine sulfate	1.1		
DL-methionine	0.2		
L-threonine	0.5		
L-tryptophan	0.1		
Vitamins	0.05		
Minerals	0.1		
Wheat bran	2.45		
Total	100		

Supplementary Table S2 Primer sequences for RT-qPCR

Gene	5'-3' Primer sequence
SLC10A2	F: CCAGAGTGCCTGGATCATCG
	R: GGAGTAACCGGCCAAAGGAA
SLC1A1	F: GTTCCTGATTGCCGGGAAGA
	R: ATGGCGAATCGGAAAGGGTT
MEP1A	F: CAACTTAGATGCAGGCTTGGAC
	R: CCATCTGGTGTTGGGGTCTC
NR1H4	F: TCAGTCCTTGTCACAGCCAC
	R: AGAGGACCTGCTGCTTGTTC
β-actin	F: TCCCTGGAGAAGAGCTACGA
	R: TGTTGGCGTAGAGGTCCTTC
